# -374 T/A RAGE Polymorphism Is Associated with Chronic Kidney Disease Progression in Subjects Affected by Nephrocardiovascular Disease

**DOI:** 10.1371/journal.pone.0060089

**Published:** 2013-04-04

**Authors:** Ivano Baragetti, Giuseppe Danilo Norata, Cristina Sarcina, Andrea Baragetti, Francesco Rastelli, Laura Buzzi, Liliana Grigore, Katia Garlaschelli, Claudio Pozzi, Alberico Luigi Catapano

**Affiliations:** 1 Nephrology and Dialysis Unit, Bassini Hospital, Cinisello Balsamo, Milan, Italy; 2 Department of Pharmacological and Biomolecular Sciences, Università degli Studi di Milano, Milan, Italy; 3 Center for the Study of Atherosclerosis, Italian Society for the Study of Atherosclerosis (SISA) Lombardia Chapter, Bassini Hospital, Cinisello Balsamo, Milan, Italy; 4 The Blizard Institute, Barts and The London School of Medicine and Dentistry, Queen’s Mary University, London, United Kingdom; 5 Multimedica IRCCS, Milano, Italy; National Cancer Institute, United States of America

## Abstract

**Background:**

Chronic kidney disease (CKD) patients present elevated advanced glycation end products (AGEs) blood levels. AGEs promote inflammation through binding to their receptor (RAGE), located on the membrane of mesangial cells, endothelial cells and macrophages. Several genetic polymorphisms influence RAGE transcription, expression and activity, including the substitution of a thymine with an adenine (T/A) in the position -374 of the gene promoter of RAGE. Our study investigates the role of -374 T/A RAGE polymorphism in CKD progression in subjects affected by nephrocardiovascular disease.

**Methods:**

174 patients (119 males (68.4%) mean age 67.2±0.88 years; 55 females (31.6%): mean age 65.4±1.50 years) affected by mild to moderate nephrocardiovascular CKD were studied. Each subject was prospectively followed for 84 months, every 6–9 months. The primary endpoint of the study was a rise of serum creatinine concentrations above 50% of basal values or end stage renal disease.

**Results:**

Carriers of the A/A and T/A genotype presented higher plasma levels of interleukin 6 (A/A 29.5±15.83; T/A 30.0±7.89, vs T/T 12.3±5.04 p = 0.01 for both) and Macrophages chemoattractant protein 1 (A/A 347.1±39.87; T/A 411.8±48.41, vs T/T 293.5±36.20, p = 0.04 for both) than T/T subjects. Carriers of the A allele presented a faster CKD progression than wild type patients (Log-Rank test: Chi square = 6.84, p = 0,03)**.** Cox regression showed that -374 T/A RAGE polymorphism (p = 0.037), albuminuria (p = 0.01) and LDL cholesterol (p = 0.038) were directly associated with CKD progression. HDL cholesterol (p = 0.022) and BMI (p = 0.04) were inversely related to it. No relationship was found between circulating RAGE and renal function decline.

**Conclusions:**

-374 T/A RAGE polymorphism could be associated with CKD progression and inflammation. Further studies should confirm this finding and address whether inhibiting RAGE downstream signalling would be beneficial for CKD progression.

## Introduction

Oxidative stress (OS) is one of the main causes associated with chronic kidney disease progression (CKD). Beyond aging, diabetes and hypertension, several mechanisms contribute the production of reactive oxygen species (H_2_O_2_, OH^−^, O^.^) in CKD, including vitamin C deficiency due to malnutrition [1], impairment of antioxidant mechanisms [2], [3]), inflammation [4] and increased levels of advanced glycation end products (AGEs), as a consequence of their impaired renal clearance [5]. The interaction between AGEs and their receptor (RAGE) located on monocytes [6], T- lymphocytes [7] and endothelial cells [8], [9], enhances NF-kB-mediated [10] cellular production of cytokines, including interleukin-1 (IL-1), interleukin 6 (IL-6), Tumor Necrosis Factor α (TNF-α) and cell adhesion molecules. These events induce OS and reduce endothelial nitric oxide synthetase activity, thus resulting in endothelial dysfunction, a hallmark of cardiovascular complications, especially in diabetic patients [11].

RAGE is present either as a transmembrane receptor or as soluble protein (sRAGE). The latter acts as a decoy for circulating AGEs thus limiting the interaction between AGEs and membrane RAGE [12]. The gene is located on chromosome 6 (6p21.32 region). The transcription of the RAGE towards the soluble form rather than the membrane anchored form depends on two different types of post-transcriptional splicing of the messenger RNA respectively, which in turn generate two types of t-RNA [13]. It is known that higher sRAGE levels exert a protective role, in fact they are related to a lower risk of microvascular complication in type 2 diabetic patients [14]. There are several polymorphisms which could influence the transcription, the alternative splicing of the m-RNA, thus influencing the ratio between membrane and soluble RAGE, or the receptor affinity for AGEs [15], [16].

A relatively frequent polymorphism consisting in a substitution of thymine with adenine (T/A) in -374 position of the gene promoter, leading in a 3 fold increase of transcriptional activity (17), was associated with protection toward the development of cardiovascular disease (T/A or A/A individuals) in both diabetic and non-diabetic individuals [17], [18], although not all studies are consistent with these findings [19], [20]. Also the association between the -374 T/A RAGE polymorphism and diabetic nephropathy is unclear. Whereas in some studies a protective role of -374 A genotype in diabetic nephropathy was showed [17], this finding was not confirmed by others [21]. Indeed two studies observed the prevalence of the A allele in patients affected by diabetic nephropathy [22], [23].

Therefore we prospectively investigated the role of this single-nucleotide polymorphism (SNP) in the decline of renal function in patients with mild to moderate kidney dysfunction.

## Materials and Methods

### Ethics Statement

This trial has been conducted according to the principles of the Declaration of Helsinki. The trial was a substudy of CHECK Trial. It was approved by the Ethics Committee of the University of Study of Milan (Ethics committee UNIMI, approved on 06-02-2001, protocol n Pr.0003). Each patient signed an informed consent before participating to the trial.

### Patients and Study Design

174 patients have been studied (119 males (68.4%): mean age 67.2±0.88 years; 55 females (31.6%): mean age 65.4±1.50 years). All subjects were outpatients chronically followed in Nephrology Division of Bassini Hospital (Cinisello Balsamo-Italy). Patients affected by mild to moderate chronic kidney dysfunction (mean GFR of 65±5.65 ml/min) were enrolled. The enrolment lasted 1 month (from 1^st^ January 2005 to 1^st^ February 2005). Subjects were divided into three groups, on the basis of their -374 T/A RAGE genotype (wild type, heterozygous for the A allele, homozygous for the A allele). Patients groups were matched for all anthropometric, clinical and biohumoral parameters. Patients affected by any inflammatory or infective pathologies, congenital or hereditary kidney diseases, glomerulonephritis, malignant neoplasia, cardiovascular events in the 6 months before (acute myocardial infarction, stroke, transient brain ischemic attacks, acute coronary syndrome, carotid thromboarterectomy, percutaneous coronary angioplasty, arterial angioplasty at the inferior limbs, coronary by-pass, arterial by-pass at the inferior limbs), major surgery in the previous 6 months, acute heart failure in the 6 months before, chronic heart failure NYHA III and IV, have been excluded from the trial. At the enrolment medical history (comprehensive of a drug history) was investigated and examined, including arterial pressure measurement (systolic, diastolic and mean blood pressure; the mean values of three measurements performed every 15 minutes were registered), weight and height (for BMI calculation) and waist circumference measurement.

Basal blood and urine samples were taken for the following laboratory tests: urea, creatinine, electrolytes, calcium, phosphorus, PTH, uric acid, glycated haemoglobin, blood glucose, haemoglobin, bicarbonates, iron assessment, albumin, lipid profile, PCR, interleukin 6, interleukin 8, macrophage chemoattractant protein 1, leptin, adiponectin, circulating RAGEs, albuminuria, urinary sodium and urinary urea and a genotyping for the –374 T/A RAGE polymorphism.

GFR was assessed both as calculated creatinine clearance (using the 24 hours urine collection) and as estimated GFR, using the Levey’s formula [24].

Each subject was prospectively followed for 84 months and visited every 6–9 months, even blood samples were taken for the routine clinical biohumoral analyses (comprehensive serum creatinine). 24 hour urine was collected and sent to our central laboratory together with blood samples every visit.

The endpoint of the study was a rise of serum creatinine plasma concentrations above 50% of the basal values or severe renal dysfunction requiring dialysis treatment in the short period.

Patients who neeeded dialysis urgently went to our observation as late referrals, having not respected the follow-up schedule.

### Laboratory Methods

Blood and urine samples were collected after over-night fasted. After centrifugation at 3.000 rpm for 12 minutes, samples were stored at −80°C.

In sera determinations of cardiometabolic markers (total cholesterol, HDL, triglycerides, and glycemia) as well as hepatic enzymes (ALT, AST, γGT, CPK), creatinine and uric acid levels were executed with colorimetric method using Cobas Mira Plus analyzer (Horiba®, ABX, France) [25].

LDL cholesterol fraction was calculated using Friedewald formula as described.

Fresh samples of blood were used for determination of: glycated haemoglobin (HPLC, %), urea (enzymatic method, mg/dL), hemoglobin (g/dL), leukocytes count (10^3^ uL), emogas-analysis for electrolytes detection, plasmatic albumin (electrophoresis, g/dL), inorganic phosphorus (molybdate test, mg/dL), parathyroid hormone (ECLIA, pg/mL), iron (ferrozine assay, ug/dL), ferritin (ECLIA, ng/mL), transferrin and C-reactive protein (immunoturbidimetric, mg/dL). IL-6, IL-8 and MCP-1 levels were assessed with BIO-PLEX™ assay (Bio-Rad, Hercules, CA, USA). Plasma adiponectin levels (all the isoforms) were measured using a commercial available ELISA kit (Assaypro, Winfield, MO, USA) whereas leptin levels in plasma samples were assessed through RIA method (Leptin Tin Human Millipore®, St. Charles, Missouri, USA) as described [26], [27].

### Genotyping

Genomic DNA was extracted using the Flexigene DNA kit (Qiagen, Milan, Italy) [28]. Genotyping for the -374 T/A RAGE polymorphism was performed on 1 *µ*L (10 to 200 ng of DNA), using a TaqMan allelic discrimination test. The primers used were the following: FW 5′- -3′, REV 5′- -3′ and the probes were FAM- -BQ1 and Texas Red- -BQ2.

### Peripheral Blood Mononuclear Cells and Macrophages mRNA Analysis

-374 T/A RAGE polymorphism was assessed with real time PCR. Briefly blood diluted 1∶3 in PBS (15 ml) was layered onto 4 ml of Ficoll Hipaque (Amersham) and centrifuged at 1500 rpm for 35 min. Peripheral blood mononuclear cells were removed from the interface and washed twice (10 min 1500 rpm) in PBS before being counted. Total RNA was extracted and underwent reverse transcription as described [29], [30]. Three µL of cDNA were amplified by real-time quantitative PCR with 1X Syber green universal PCR mastermix (BioRad). The specificity of the Syber green fluorescence was tested by plotting fluorescence as a function of temperature to generate a melting curve of the amplicon. The primers used are described elsewhere [31], [32]. The melting peaks of the amplicons were as expected (not shown). Each sample was analyzed in duplicate using the IQ-Cycler (BioRad). The PCR amplification was related to a standard curve ranging from 10^−11^ M to 10^−14^ M.

### sRAGE Levels Determination

sRAGE levels were determined via ELISA assay using Quantikine® Human RAGE kit (R&D System, Minneapolis, USA) as described [33]. Briefly, pre-coated microplates with monoclonal antibody specifics for RAGE’s extracellular domain were used. Firstly, 100 µL of Assay Diluent, 50 µL of sample (plasma stored at −20°C) and 50 µL of RAGE standard solutions (5000 pg/mL–2500 pg/mL–1250 pg/mL–625 pg/mL–312 pg/mL–156 pg/mL–78 pg/mL) were added. After two hours of incubation period at 25°C, the content of each well was aspired and four wash cycles with Wash Buffer solution were made. Then, the RAGE conjugate was added to react with the antibody and the microplate was exposed to two hours of incubation at 25°C. After another cycle of washes, Substrate Solution was added and the colour developed proportionally to the amount of RAGE bound in the initial step. Finally, Stop Solution (H_2_SO_4_ 2 N) was added to stop the reaction and via spectrometer lecture was made at 450 nm. Results were expressed as pg/mL.

### Left Ventricular Mass was Evaluated with Echocardiography

Echocardiograms were performed at rest with patients supine in the left lateral side, using standard parasternal and apical views. The overall monodimensional left ventricular measurements and the bidimensional (apical four and two chamber) views have been obtained according to the recommendations of the American Society of Echocardiography. All tracings have been done and read by a single observer blinded to the clinical characteristics of the patients under observation. LV mass has been derived using the formula described by Devereux and colleagues [34]:

LV Mass (grams) = 0.80×1.04 [(VSTd+LVIDd+PWTd)^3^−(LVIDd)^3^]+0.6, where VSTd is ventricular septal thickness at end diastole, LVIDd is LV internal dimension at end diastole, and PWTd is LV posterior wall thickness at end diastole. Left ventricular mass has been corrected for height ^2.7^ (LVMI), and expressed in units of grams/meter (g/m^2.7^). The presence of left ventricular hypertrophy (LVH) has been defined for LVMI>51 g/m^2.7^ in either gender.

### Carotid Intima-media Thickness

Intima plus media thickness (IMT) of both carotid arteries has been evaluated by high resolution US scan, Biosound 2000 SA (Minneapolis, In, USA) with a 8-MHz transducer as described [35]. Carotid artery has been scanned at the internal, at the bifurcation and at the common carotid artery (CCA). At each longitudinal projection the far-wall IMT, as defined by Wendelhag [36], was measured in five standardized points, in the first centimetre proximal to the bulb dilation. Carotid plaque has been defined as IMT>1.3 mm. IMT has been measured on CCA outside the plaque, if any was present. Each patient’s IMT has been calculated taking the averages of ten measurements, 5 in the left and 5 in the right carotid artery.

### Endothelial Functionality Assessment – Flow-Mediated Dilatation

Endothelial function was evaluated non-invasively by B-mode ultrasonography (SA 6000C-MT, Medison, South Korea) as described elsewhere [31]. Briefly, each subject was requested to lie at rest for 10 min in a temperature-controlled room (21°C±1), and the first scan of brachial artery in the left arm was taken.

This was followed by inflation of a standard pneumatic tourniquet placed around the upper arm at a pressure of 200 mmHg. After cuff removal, electrocardiography was monitored continuously during the study and measurements were taken at the end diastole.

Vessel images were taken at rest and during reactive hyperemia: FMD was calculated 90–210 sec after the deflation of a pneumatic tourniquet. NMD was calculated as the percentage in variation between the basal diameter and the maximum diameter after sublingual administration or glyceryl trinitrate 0.3 mg.

### Statistical Analysis

Statistical analysis was performed using the statistical package STATA/SE 9.2 for Windows XP. Results of the continuous variables were expressed as Mean ± Standard Error.

The three groups of patients (T/T, T/A and A/A -374 RAGE genotypes) were compared each other in terms of anthropometric, clinical, instrumental and biohumoral parameters using a one-way ANOVA. The post Hoc analysis was performed using the Bonferroni’s Test. The distribution of sexes, diabetes, medications, smoke habitude among the three groups was assessed with a Chi square. The significance was assumed for p values <0.05.

The survival analysis was performed using the Kaplan Mayer method. The rate of survival was compared between the three groups of patients using the Log-Rank test. The significance was assumed for p values <0.05.

Finally two separate Cox multivariate models were generated including in the analysis the -374 T/A RAGE genotype, sRAGE and all the principal variables of nephrological interest as covariates and the rising of serum creatinine concentrations or dialysis as the outcome variable. The Enter method was used. The significance was assumed for p values <0.05.

## Results

The anthropometric and biohumoral characteristics of patients enrolled in the trial are shown in table 1. The -374 T/A RAGE distribution was in Hardy-Weinberg equilibrium with 31.6% of patients having the T/T genotype, 50.0% the T/A genotype and 18.4% the A/A genotype.

**Table 1:** Baseline characteristics of the population according to genotype.

	RAGE T/T	RAGE T/A	RAGE A/A	Chi square	p
**Sex (n° M/F)**	35/20	62/25	22/10	0.90	0.63
**Diabetes (n°- %)**	40 (72.7)	60 (69.0)	22 (68.8)	0.26	0.87
**Smoke (n°- %)**	9 (16.4)	18 (20.7)	7 (21.9)	0.53	0.76
**Past cardiovascular events** **(n°-%)**	8 (14.5)	23 (26.4)	8 (25)	2.89	0.23
				F test	p
**Age (years)**	64,9±1,22	67,3±1,19	67.5±1.91	1.0	0,37
**BMI (Kg/m^2^)**	29,2±0,77	29,2±0.65	29.4±1.03	0.01	0.98
**Waist circumference (cm)**	103.5±1,96	102.6±1,63	105.1±2.63	0.87	0.41
**Mean arterial pressure (mmHg)**	107.5±2.57	102.1±1.30	101.1±1.89	2.98	0.054
**PTH (pg/mL)**	98.7±21.27	72.5±11.13	77.3±11.68	0.85	0,42
**Hemoglobin (g/dL)**	13.3±0.23	13.1±0.22	13.3±0.30	0.23	0.79
**Glycated Hemoglobin (%)**	6.7±0.20	6.9±0.77	6.9±0.21	0.75	0.47
**Uric acid (mg/dL)**	6.1±0.20	6.7±0.54	6.4±0.27	0.34	0.71
**Urea (mg/dL)**	57.2±4.47	66.1±4.84	58.8±5.86	0.95	0.38
**Creatinine (mg/dL)**	1.48±0.14	1.6±0.12	1.4±0.12	0.47	0.62
**eGFR (mL/min)**	67.7±5.43	57.2±3.62	65.0±7.30	1.41	0.24
**Calculated GFR (mL/min)**	70.3±9.42	59.2±4.91	58.1±12.29	0.73	0.48
**Na (mmol/L)**	134.6±0.76	133.9±0.06	134.4±0.09	0.29	0.74
**K (mmol/L)**	4.3±0.10	4.3±0.07	4.2±0.11	0.56	0.56
**Ca x P product (mg/dL)**	29.5±1.30	30.3±1.18	28.1±1.69	0.57	0.56
**Serum Bicarbonates (mmol/L)**	26.6±0.61	25.9±0.36	27.3±0.61	1.77	0.17
**Serum Iron (µg/dL)**	55.7±5.62	59.7±3.59	47.9±7.13	1.26	0.28
**Transerrin (mg/dL)**	244±10.02	255.6±6.26	264.2±14.2	0.89	0.41
**Ferritin (ng/mL)**	175±26.06	161.3±21.61	116.6±20.65	1.03	0.36
**Albumin (g/dL)**	4.3±0.04	4.2±0.04	4.2±0.10	1.49	0.22
**Total cholesterol (mg/dL)**	19.9±6.25	203.7±5.22	207.8±7.36	0.59	0.55
**HDL cholesterol (mg/dL)**	52±2.40	48.4±1.37	50.8±2.57	1.04	0.35
**LDL cholesterol (mg/dL)**	117.2±5.59	122.9±4.67	126.4±7.32	0.53	0.58
**Triglycerides (mg/dL)**	40.5±4.26	38.1±3.33	38.0±5.59	0.11	0.89
**24 h urinary sodium (mmol/24 h)**	164.7±11.63	162.6±8.10	182.2±19.25	0.63	0.53
**24 h urinary urea (g/24 h)**	2.0±0.98	2.1±1.02	2.4±0.37	1.01	0.36
**CRP (mg/dL)**	0.33±0.067	0.30±0.055	0.44±0.165	0.59	0.55
**Albuminuria (mg urinary albumin/mmol urinary creatinine**	43.7±13.83	73.1±16.37	24.3±9.78	2.22	0.11
**LVM (g/h^2.7^ )**	50.6±2.45	53.2±1.89	57.2±3.78	1.29	0.27
**Carotid Intima-Media Thickness (mm)**	0.78±0.029	0.79±0.025	0.76±0.031	0.12	0.88
**Flow mediated brachial artery dilation (%)**	13.6±1.08	11.2±0.84	15.3±3.07	2.11	0.12
**Adiponectin (µg/mL)**	18.2±1.46	19.5±1.32	18.2±2.17	0.27	0.76
**Leptin (ng/mL)**	19.9±3.82	20.0±2.18	19.0±4.14	0.02	0.97
**Serum RAGE (pg/mL)**	1633.8±137.22	1950.7±108.7	1626.8±121.1	2.42	0.09
**Interleukin 6 (pg/mL)**	12.3±5.04	30.0±7.89	29.5±15.83	1.13	0.01
**Interleukin 8 (mg/dL)**	70.1±24.98	47.5±10.44	56.1±19.77	0.46	0.63
**Macrophages chemoactrant** **protein 1 (pg/mL)**	293.5±36.20	411.8±48.41	347.1±39.87	1.63	0.04

The anthropometric parameters, the prevalence of past cardiovascular events (myocardial infarction, acute coronary syndrome, stroke, transient ischemic attacs, by-passes at the inferior limbs, angioplasty, aorto-coronary by-passes) and biohumoral parameters are compared between patients carrying the –374 T/T,T/A and A/A genotypes of RAGE. Significance have been taken for p values <0.05, using a single way ANOVA: the post-hoc analysis showed a statistically significant difference between T/T and T/A subjects vs A/A subjects in terms of Interleukin 6 and Macrophages chemoattractant protein 1. No differences have been seen in terms of renal function, albuminuria, intermediate cardiovascular organ damage, inflammatory parameters or nutritional parameters between the three groups of subjects.

No significant differences among the genotypes were observed according to age, sex, gender, mean arterial pressure, presence of diabetes, BMI, waist circumference, smoking habits and previous cardiovascular events (table 1).

The same was true for kidney and metabolic function; indeed electrolytes assessments, calcium-phosphorus metabolism, glycometabolic control, nutritional parameters, uric acid, hemoglobin, iron assessment, bicarbonates serum concentrations, total cholesterol, HDL and LDL cholesterol, triglycerides, PCR, interleukin 8, adiponectin and leptin plasma concentrations were similar among the genotypes (Table 1). Carries of the A allele showed significantly higher plasma levels of interleukin 6 (T/T: 12.3±5.04, T/A: 30.0±7.89, A/A: 29.5±15.83, respectively; p<0.01: T/A and A/A vs T/T ) and macrophages chemoattractant protein 1 (T/T: 293.5±36.20, T/A: 411.8±48.41, A/A: 347.1±39.87, respectively; p = 0.04: T/A and A/A vs T/T) compared to T/T subjects while plasma levels of sRAGE were only slightly increased (T/T: 1633.8±137.22 pg/mL, T/A: 1950.7±108.7 and A/A: 1626.8±121.1 pg/mL, p = 0,09).

The presence of the -374 T/A SNP was not associated with subclinical cardiovascular disease, in fact carotid intima-media thickness, flow mediated brachial artery vasodilation and left ventricular mass were not statistically different among the RAGE genotypes. Finally the distribution of anti-hypertensive, anti-diabetic or lipid-lowering therapies was similar among the three groups (table 2).

**Table 2:** Prevalence of medications according to genotypes.

Medication yes/no (n°- % of all patients studied)	RAGE T/T (n° = 55)	RAGE T/A (n° = 87)	RAGE A/A (n° = 32)	Chi square	p
**ASA/Ticlopidine**	22/33(40.0)	37/50 (42.5)	18/14(56.3)	2.37	0.30
**Allopurinol**	12/43(21.8)	14/73(16.1)	4/28(12.5)	1.39	0.49
**Statins or fibrates**	20/35(36.4)	28/59 (32.2)	16/16(50.0)	3.2	0.20
**β blockers or αβ Blockers**	10/45(18.2)	20/67 (23.0)	6/26(18.8)	0.56	0.75
**Clonidine**	0/55 (0)	3/84 (3.4)	1/31(3.1)	1.90	0.38
**α antagonists**	2/53 (3.6)	11/76(12.6)	2/30(6.3)	3.75	0.15
**Calcium channel blockers (DDP and NDDP)**	10/45(18.2)	26/61(29.9)	10/22(31.3)	2.84	0.24
**Diuretics**	18/37(32.7)	32/55 (36.8)	13/19 (40.6)	0.57	0.75
**ARBs**	21/34(38.2)	26/61 (29.9)	10/22 (31.3)	1.09	0.57
**ACE inhibitors**	28/27(50.9)	36/51 (41.4)	17/15 (53.1)	1.91	0.38
**Oral antidiabetics**	27/28 (49)	33/54 (37.9)	15/17 (46.9)	1.93	0.37
**Insulin**	7/48 (12.7)	19/68 (21.8)	2/30(6.3)	4.88	0.09

Medications: no differences have been found between patients carrying –374 T/T, T/A and A/A genotypes in terms of antihypertensive or antidiabetic therapy. A Pearson Chi square test was used, keeping a significant difference for p values <0.05.

During the follow-up period we observed 40 events comprehensive of the serum creatinine increase of more than 50% of the basal values and need of dialysis (22%).

**Figure 1:**
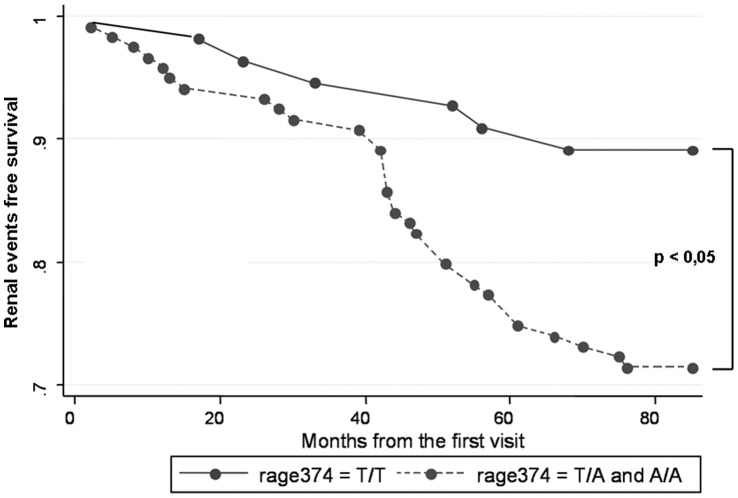
Renal survival of patients carrying –374 T/T and the A allele. The figure shows that the subjects carrying the A allele present a faster decline of renal function than wild type patients. The main endpoint of the analysis was an increase of serum creatinine over 50% or the beginning of chronic dialysis. The figure shows a total of 40 events: 6 in T/T subjects, 34 in subjects carrying the A allele.

Survival analysis showed a faster decline of renal function in carriers of the A allele compared to TT subjects **(**Log-Rank test: Chi square = 6.34, p = 0,018) (fig. 1).

**Figure 2:**
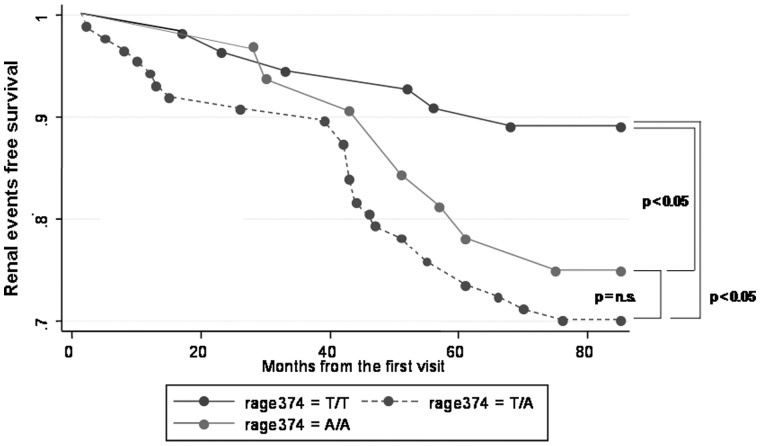
Renal survival of wild-type, heterozygous and homozygous patients for the A allele. The figure shows that T/A and A/A subjects present a faster decline of renal function than T/T patients. The main end point of the analysis was an increase of serum creatinine over 50% or the beginning of chronic dialysis. Figure shows a total of 40 events: 6 in T/T subjects, 26 in T/A subjects and 8 in A/A subjects.

Differences in renal survival were also maintained when the three genotypes were analyzed independently; with a significant difference in terms of survival between T/T subjects and T/A and A/A subjects (Log-Rank test: Chi square = 6.84, p = 0,03) (fig. 2).

Overall T/T subjects presented a mean percentage of endpoint achievement of 89±0.04% at 67 months, those belonging to T/A group showed a mean percentage of endpoint achievement 70±0.04% at 75 months and patients carrying the A/A genotype 75±0.07% at 74 months.

Furthermore multivariate analysis was then performed to identify among albuminuria, LDL-cholesterol, HDL-cholesterol, -374 T/A RAGE, BMI, GFR, mean arterial pressure, haemoglobin levels and calcium-phosphorus product, the main predictors of renal function decline (Table 3). As expected, albuminuria resulted the main predictor [Wald test 6.550 and a mean Hazard ratio of 1.015 (increased risk of reaching the end point of 1.5% for each mg/l in more of albuminuria) (IC 95%: 1.003–1.025), p = 0.01] followed by the-374 RAGE polymorphism [Wald test of 4.330 and a mean Hazard ratio of 2.724 (increased risk of reaching the end point of 2.724 fold higher for subjects carrying the A allele than those carrying T/T genotype) (IC 95%: 1.060–6.998), p = 0.037], LDL cholesterol [Wald test of 4.310 and an Hazard ratio of 1.009 (increased risk of reaching the end point of 0.9% per each mg/dl increase of LDL cholesterol) (IC 95%:.1.000–1.017, p = 0.038)], HDL cholesterol [Wald test of 5.253 and an Hazard Ratio of 0.958 (decreased risk of reaching the end point of 5% per each mg/dl in more of HDL cholesterol) (IC 95%: 0.941–0.995, p = 0.022)] and BMI [Wald test of 4.215 and an Hazard ratio of 0.933 (decreased risk of reaching the end point of 0.7% per each Kg/m^2^ in more of BMI) (IC 95%: 0.873–0.997, p = 0.040)].

**Table 3:** Cox regression for the decline of renal function including -374 T/A RAGE.

Covariates	Beta	Beta Standard Error	wald	p	Hazard Ratio	CI Hazard ratio
Haemoglobin (g/dL)	−0.156	0.114	1.854	0.172	0.855	0.584–1.070
GFR (mL/min)	−0.005	0.007	0.477	0.490	0.995	0.982–1.009
Albuminuria (mg/L)	0.015	0.006	6.550	0.01	1.015	1.003–1.025
Mean arterial pressure (mmHg)	0.21	0.015	2.011	0.153	1.021	0.992–1.051
-374 A RAGE	1.002	0.481	4.330	0.037	2.724	1.060–6.998
Ca x P product (mg/dL)	0.059	0.032	3.285	0.070	1.060	0.995–1.130
HDL Cholesterol (mg/dL)	−0.033	0.014	5.253	0.022	0.958	0.941–0.995
LDL Cholesterol (mg/dL)	0.008	0.004	4.310	0.038	1.009	1.000–1.017
BMI (Kg/m^2^)	−0.059	0.034	4.215	0.040	0.933	0.873–0.997

Cox regression. Table shows that –374 A RAGE genotype, together with albuminuria, LDL cholesterol, HDL cholesterol and BMI are significantly associated with the decline of renal function. –374 A allele for RAGE, albuminuria and LDL cholesterol are predictor of CKD progression, while HDL cholesterol and BMI are inversely associated with renal function decline.

In the second multivariate model including albuminuria, LDL-cholesterol, HDL-cholesterol, sRAGE, BMI, GFR, mean arterial pressure, haemoglobin levels and calcium-phosphorus product, only albuminura [Wald test 6.5 and a mean Hazard ratio of 1.016 (increased risk of reaching the end point of 1.6% for each mg/l in more of albuminuria) (IC 95%: 1.004–1.029), p = 0.01] followed by HDL cholesterol [Wald test of 5.26 and a mean Hazard ratio of 0.97 (reduced risk of reaching the end point of 3% per each mg/dl in more of HDL cholesterol) (IC 95%: 0.94–0.99), p = 0.031] predictors of CKD progression (Table 4).

**Table 4:** Cox regression for the decline of renal function including the levels of the soluble form of RAGE.

Covariates	Beta	Beta Standard Error	wald	p	Hazard Ratio	CI Hazard ratio
Hemoglobin (g/dL)	−0.145	0.112	1.850	0.176	0.841	0.656–1.080
GFR (mL/min)	−0.001	0.004	0.423	0.948	1.004	0.987–1.013
Albuminuria (mg/L)	0.013	0.005	6.500	0.010	1.016	1.004–1.029
Mean arterial pressure (mmHg)	0.20	0.012	2.014	0.080	1.019	0.998–1.042
Tertiles sRAGE (pg/mL)	−0.003	0.005	0.530	0.590	1.001	0.991–1.005
Ca x P product (mg/dL)	0.061	0.038	3.310	0.185	1.045	0.978–1.116
HDL Cholesterol (mg/dL)	−0.034	0.014	5.260	0.031	0.969	0.942–0.997
LDL Cholesterol (mg/dL)	0.075	0.048	2.102	0.105	1.007	0.998–1.016
BMI (Kg/m^2^)	−0.118	0.015	2.015	0.099	0.938	0.861–1.012

Cox regression. Table 3b shows that replacing sRAGE rather than –374 A RAGE genotype in the same model showed in table 3a, only albuminuria, and HDL cholesterol are significantly associated with the decline of renal function.

## Discussion

Our results show that CKD patients with the –374 RAGE A allele have a poor CKD prognosis compared to carriers of the TT genotype. Although the role of –374 T/A RAGE in CKD progression has not been extensively investigated, some cross-sectional studies showed an association of the A allele with CKD in diabetic subjects [22], [23], in contrast with the protective role exerted by this allele toward cardiovascular disease [21]. To the best of our knowledge, our trial is the first which prospectively shows a direct relationship between –374 T/A RAGE polymorphism and the decline of kidney function in patients with cardiovascular renal disease. Of note, a recent meta-analysis showed that other SNPs known to affect RAGE transcription are not associated with the prevalence of diabetic nephropathy [37] further supporting the relevance of our finding.

This finding suggests that the presence of the T allele is protective toward the decline of kidney function compared to a potential protective effect of the A allele in cardiovascular disorders showed in previous studies [21]. The molecular mechanisms behind this effect need further investigations. The presence of the -374 A allele is associated with increased RAGE transcription [16]; however, while the increase in RAGE plasma levels observed in carriers of the A allele was not statistically significant we cannot exclude that specific isoforms of RAGE are differently affected. Indeed, the transcription of the RAGE towards the soluble form rather than the membrane form depends on a different post-transcriptional splicing of the messenger RNA (38). It is known that higher sRAGE levels exert a protective role, in fact they are related to a lower risk of microvascular complication in type 2 diabetic patients [14]. However sRAGE levels are contributed both by the original spliced isoforms but also by the cleaved membrane receptor [38], with potentially different roles. Of note, in vitro experiments have shown that AGEs-RAGE interaction is involved in the progression of renal damage by inducing mesangial fibrosis, glomerular sclerosis and the expression of vascular endothelial grow factor (VEGF) and MCP1 by mesangial cells [39], [40], which in turn could support monocyte mesangial infiltration in early phase of diabetic nephropathy [41]. AGEs-RAGE interaction stimulates mesangial cells production of insulin grow factor-1 and 2, platelet derived grow factor (PDGF) and transforming grow factor–β (TGF-β), which further promote mesangial production of type IV collagen, laminin and fibronectin [39]–[41]. Moreover AGEs-RAGE interaction also increases TGF- β expression by podocytes and proximal tubular cells, leading to glomerulosclerosis and tubule-interstitial fibrosis [42]. Furthermore in vivo experiments in rats showed that infusion of AGE-albumin induced glomerular hypertrophy, overexpression of type IV collagen, laminin B1 and TGF-β [42], [43], [44]. AGEs-RAGE interaction and also other metabolic factors such as HDL also increases TGF-β expression by podocytes and proximal tubular cells, leading to glomerulosclerosis and tubule-interstitial fibrosis [45], [46]. All these aspects clearly point to an involvement of the AGEs-RAGE in CKD progression. AGEs promote inflammation [47] by binding RAGE on the surface of macrophages, lymphocytes, endothelial cells and mesangial cells; the observation that in our cohort carriers of the A allele present increased levels of MCP-1 and IL-6 could support the concept of an higher inflammatory mediated kidney function decline in A carriers.

Cox analysis showed that also albuminuria and LDL cholesterol were independent predictors of CKD progression in agreement with the CKD protective effects of therapies aimed at improving proteinuria such as RAS inhibitors or lipid profile [48] such as HMGCoA reductase inhibitors [49].

The Cox analysis also showed an inverse relationship between BMI and CKD progression. Although this finding could seem somehow confounding, given that BMI, an obesity marker as waist circumference, is a risk factor for the development and the progression of chronic renal dysfunction [50], our data support the hypothesis that BMI could inversely reflect patient’s lean mass and malnutrition, which is highly prevalent among CKD patients at risk of progression [51].

We have to acknowledge some limitations of our study: firstly, we enrolled patients who already presented some degree of renal dysfunction which could result in minor differences among the genotypes. However, the long follow-up (84 months) allowed to appreciate prospectively the association of the A allele to kidney function decline compared to that of TT.

Secondly, we investigated a relatively limited number of CKD patients, however the frequencies of the -374T/A genotypes are similar to those reported in larger cohorts [18], [20], thus suggesting that our findings could set the stage for further confirmation in larger CKD cohorts. We cannot exclude that studying prospectively a cohort of subjects, all with a normal renal function and a longer follow-up could result in additional predictors.

In conclusion, our data support the role of AGEs-RAGE system in the progression of chronic renal dysfunction and suggest a potential target to further improve the management of CKD progression toward dialysis given that the conventional strategies are not sufficiently effective. Future studies addressing markers of inflammation and of angiogenesis are needed to clarify the pathophysiological mechanisms of AGEs-RAGE system activation and the association between the chronic inflammatory status and the consecutive kidney remodelling in relation to the RAGE status.

## References

[1] Locatelli F, Canaud B, Eckardt KU, Stenvinkel P, Wanner C, et al (2003) Oxidative stress in end-stage renal disease: an emerging threat to patient outcome. Nephrol Dial Transplant 18: 1272–1280. 12808161 10.1093/ndt/gfg074

[2] Mimic-Oka J, Simic T, Ekmescic V, Dragicevic P (1995) Erythrocyte glutathione peroxidase and superoxide dismutase activities in different stages of chronic renal failure. Clin Nephrol 44: 44–48. 7554532

[3] Vaziri ND, Dicus M, Ho ND, Boroujerdi-Rad L, Sindhu RK (2003) Oxidative stress and dysregulation of superoxide dismutase and NADPH oxidase in renal insufficiency. Kidney Int 63: 179–185. 12472781 10.1046/j.1523-1755.2003.00702.x

[4] Cachofeiro V, Goicochea M, de Vinuesa SG, Oubina P, Lahera V, et al.. (2008) Oxidative stress and inflammation, a link between chronic kidney disease and cardiovascular disease. Kidney Int Suppl: S4–9. 10.1038/ki.2008.516 19034325

[5] Bohlender JM, Franke S, Stein G, Wolf G (2005) Advanced glycation end products and the kidney. Am J Physiol Renal Physiol 289: F645–659. 16159899 10.1152/ajprenal.00398.2004

[6] Kirstein M, Aston C, Hintz R, Vlassara H (1992) Receptor-specific induction of insulin-like growth factor I in human monocytes by advanced glycosylation end product-modified proteins. J Clin Invest 90: 439–446. 1322940 10.1172/JCI115879 PMC443119

[7] Imani F, Horii Y, Suthanthiran M, Skolnik EY, Makita Z, et al (1993) Advanced glycosylation endproduct-specific receptors on human and rat T-lymphocytes mediate synthesis of interferon gamma: role in tissue remodeling. J Exp Med 178: 2165–2172. 8245789 10.1084/jem.178.6.2165 PMC2191269

[8] Rashid G, Benchetrit S, Fishman D, Bernheim J (2004) Effect of advanced glycation end-products on gene expression and synthesis of TNF-alpha and endothelial nitric oxide synthase by endothelial cells. Kidney Int 66: 1099–1106. 15327404 10.1111/j.1523-1755.2004.00860.x

[9] Bucala R, Tracey KJ, Cerami A (1991) Advanced glycosylation products quench nitric oxide and mediate defective endothelium-dependent vasodilatation in experimental diabetes. J Clin Invest 87: 432–438. 1991829 10.1172/JCI115014 PMC295094

[10] Yamagishi S, Nakamura K, Matsui T, Noda Y, Imaizumi T (2008) Receptor for advanced glycation end products (RAGE): a novel therapeutic target for diabetic vascular complication. Curr Pharm Des 14: 487–495. 18289075 10.2174/138161208783597416

[11] Widlansky ME, Gokce N, Keaney JF Jr, Vita JA (2003) The clinical implications of endothelial dysfunction. J Am Coll Cardiol 42: 1149–1160. 14522472 10.1016/s0735-1097(03)00994-x

[12] Falcone C, Emanuele E, D’Angelo A, Buzzi MP, Belvito C, et al (2005) Plasma levels of soluble receptor for advanced glycation end products and coronary artery disease in nondiabetic men. Arterioscler Thromb Vasc Biol 25: 1032–1037. 15731496 10.1161/01.ATV.0000160342.20342.00

[13] Ramasamy R, Yan SF, Schmidt AM (2009) RAGE: therapeutic target and biomarker of the inflammatory response–the evidence mounts. J Leukoc Biol 86: 505–512. 19477910 10.1189/jlb.0409230

[14] Grossin N, Wautier MP, Meas T, Guillausseau PJ, Massin P, et al (2008) Severity of diabetic microvascular complications is associated with a low soluble RAGE level. Diabetes Metab 34: 392–395. 18701333 10.1016/j.diabet.2008.04.003

[15] Yan SF, Ramasamy R, Schmidt AM (2010) The RAGE axis: a fundamental mechanism signaling danger to the vulnerable vasculature. Circ Res 106: 842–853. 20299674 10.1161/CIRCRESAHA.109.212217 PMC2862596

[16] Hudson BI, Stickland MH, Futers TS, Grant PJ (2001) Effects of novel polymorphisms in the RAGE gene on transcriptional regulation and their association with diabetic retinopathy. Diabetes 50: 1505–1511. 11375354 10.2337/diabetes.50.6.1505

[17] Pettersson-Fernholm K, Forsblom C, Hudson BI, Perola M, Grant PJ, et al (2003) The functional -374 T/A RAGE gene polymorphism is associated with proteinuria and cardiovascular disease in type 1 diabetic patients. Diabetes 52: 891–894. 12606536 10.2337/diabetes.52.3.891

[18] Falcone C, Geroldi D, Buzzi MP, Emanuele E, Yilmaz Y, et al (2008) The -374T/A RAGE polymorphism protects against future cardiac events in nondiabetic patients with coronary artery disease. Arch Med Res 39: 320–325. 18279705 10.1016/j.arcmed.2007.11.003

[19] Kucukhuseyin O, Aydogan HY, Isbir CS, Isbir T (2009) Associations of -374T/A polymorphism of receptor for advanced glycation end products (RAGE) gene in Turkish diabetic and non-diabetic patients with coronary artery disease. In Vivo 23: 949–954. 20023238

[20] Kirbis J, Milutinovic A, Steblovnik K, Teran N, Terzic R, et al (2004) The -429 T/C and -374 T/A gene polymorphisms of the receptor of advanced glycation end products gene (RAGE) are not risk factors for coronary artery disease in Slovene population with type 2 diabetes. Coll Antropol 28: 611–616. 15666591

[21] dos Santos KG, Canani LH, Gross JL, Tschiedel B, Pires Souto KE, et al (2005) The -374A allele of the receptor for advanced glycation end products gene is associated with a decreased risk of ischemic heart disease in African-Brazilians with type 2 diabetes. Mol Genet Metab 85: 149–156. 15896660 10.1016/j.ymgme.2005.02.010

[22] Lindholm E, Bakhtadze E, Sjogren M, Cilio CM, Agardh E, et al (2006) The -374 T/A polymorphism in the gene encoding RAGE is associated with diabetic nephropathy and retinopathy in type 1 diabetic patients. Diabetologia 49: 2745–2755. 16969646 10.1007/s00125-006-0412-3

[23] Abdel-Azeez HA, El-Okely AM (2009) Association of the receptor for advanced glycation end products (RAGE) -374 T/A gene polymorphism and circulating soluble RAGE with nephropathy in type 1 diabetic patients. Egypt J Immunol 16: 95–106. 20726326

[24] Goolsby MJ (2002) National Kidney Foundation Guidelines for chronic kidney disease: evaluation, classification, and stratification. J Am Acad Nurse Pract 14: 238–242. 12087782 10.1111/j.1745-7599.2002.tb00119.x

[25] Norata GD, Baragetti I, Raselli S, Stucchi A, Garlaschelli K, et al (2010) Plasma adiponectin levels in chronic kidney disease patients: relation with molecular inflammatory profile and metabolic status. Nutr Metab Cardiovasc Dis 20: 56–63. 19359150 10.1016/j.numecd.2009.01.011

[26] Norata GD, Garlaschelli K, Grigore L, Raselli S, Tramontana S, et al (2010) Effects of PCSK9 variants on common carotid artery intima media thickness and relation to ApoE alleles. Atherosclerosis 208: 177–182. 19619878 10.1016/j.atherosclerosis.2009.06.023

[27] Norata GD, Raselli S, Grigore L, Garlaschelli K, Dozio E, et al (2007) Leptin:adiponectin ratio is an independent predictor of intima media thickness of the common carotid artery. Stroke 38: 2844–2846. 17823381 10.1161/STROKEAHA.107.485540

[28] Predazzi IM, Norata GD, Vecchione L, Garlaschelli K, Amati F, et al (2012) Association between OLR1 K167N SNP and Intima Media Thickness of the Common Carotid Artery in the General Population. PLoS One 7: e31086. 22347434 10.1371/journal.pone.0031086 PMC3276570

[29] Norata GD, Garlaschelli K, Ongari M, Raselli S, Grigore L, et al (2006) Effects of fractalkine receptor variants on common carotid artery intima-media thickness. Stroke 37: 1558–1561. 16675737 10.1161/01.STR.0000221803.16897.22

[30] Ammirati E, Cianflone D, Banfi M, Vecchio V, Palini A, et al (2010) Circulating CD4+CD25hiCD127lo Regulatory T-Cell Levels Do Not Reflect the Extent or Severity of Carotid and Coronary Atherosclerosis. Arterioscler Thromb Vasc Biol 30: 1832–1841. 20539016 10.1161/ATVBAHA.110.206813

[31] Norata GD, Grigore L, Raselli S, Redaelli L, Hamsten A, et al (2007) Post-prandial endothelial dysfunction in hypertriglyceridemic subjects: molecular mechanisms and gene expression studies. Atherosclerosis 193: 321–327. 17055512 10.1016/j.atherosclerosis.2006.09.015

[32] Norata GD, Raselli S, Grigore L, Garlaschelli K, Vianello D, et al (2009) Small dense LDL and VLDL predict common carotid artery IMT and elicit an inflammatory response in peripheral blood mononuclear and endothelial cells. Atherosclerosis 206: 556–562. 19376517 10.1016/j.atherosclerosis.2009.03.017

[33] Norata GD, Garlaschelli K, Grigore L, Tibolla G, Raselli S, et al (2009) Circulating soluble receptor for advanced glycation end products is inversely associated with body mass index and waist/hip ratio in the general population. Nutr Metab Cardiovasc Dis 19: 129–134. 18595673 10.1016/j.numecd.2008.03.004

[34] Devereux RB, Alonso DR, Lutas EM, Gottlieb GJ, Campo E, et al (1986) Echocardiographic assessment of left ventricular hypertrophy: comparison to necropsy findings. Am J Cardiol 57: 450–458. 2936235 10.1016/0002-9149(86)90771-x

[35] Ammirati E, Cianflone D, Vecchio V, Banfi M, Vermi AC, et al (2012) Effector Memory T cells Are Associated With Atherosclerosis in Humans and Animal Models. J Am Heart Assoc 1: 27–41. 23130116 10.1161/JAHA.111.000125 PMC3487313

[36] Wendelhag I, Wiklund O, Wikstrand J (1993) Atherosclerotic changes in the femoral and carotid arteries in familial hypercholesterolemia. Ultrasonographic assessment of intima-media thickness and plaque occurrence. Arterioscler Thromb 13: 1404–1411. 8399076 10.1161/01.atv.13.10.1404

[37] Kang P, Tian C, Jia C (2012) Association of RAGE gene polymorphisms with type 2 diabetes mellitus, diabetic retinopathy and diabetic nephropathy. Gene 500: 1–9. 22475522 10.1016/j.gene.2012.03.056

[38] Kalea AZ, Schmidt AM, Hudson BI (2011) Alternative splicing of RAGE: roles in biology and disease. Front Biosci 16: 2756–2770. 10.2741/3884 21622207

[39] Wendt TM, Tanji N, Guo J, Kislinger TR, Qu W, et al (2003) RAGE drives the development of glomerulosclerosis and implicates podocyte activation in the pathogenesis of diabetic nephropathy. Am J Pathol 162: 1123–1137. 12651605 10.1016/S0002-9440(10)63909-0 PMC1851245

[40] Ehlermann P, Eggers K, Bierhaus A, Most P, Weichenhan D, et al (2006) Increased proinflammatory endothelial response to S100A8/A9 after preactivation through advanced glycation end products. Cardiovasc Diabetol 5: 6. 16573830 10.1186/1475-2840-5-6 PMC1475836

[41] Yamagishi S, Fukami K, Ueda S, Okuda S (2007) Molecular mechanisms of diabetic nephropathy and its therapeutic intervention. Curr Drug Targets 8: 952–959. 17691932 10.2174/138945007781386884

[42] Fukami K, Ueda S, Yamagishi S, Kato S, Inagaki Y, et al (2004) AGEs activate mesangial TGF-beta-Smad signaling via an angiotensin II type I receptor interaction. Kidney Int 66: 2137–2147. 15569303 10.1111/j.1523-1755.2004.66004.x

[43] Yamagishi S, Inagaki Y, Okamoto T, Amano S, Koga K, et al (2003) Advanced glycation end products inhibit de novo protein synthesis and induce TGF-beta overexpression in proximal tubular cells. Kidney Int 63: 464–473. 12631112 10.1046/j.1523-1755.2003.00752.x

[44] Ziyadeh FN, Hoffman BB, Han DC, Iglesias-De La Cruz MC, Hong SW, et al (2000) Long-term prevention of renal insufficiency, excess matrix gene expression, and glomerular mesangial matrix expansion by treatment with monoclonal antitransforming growth factor-beta antibody in db/db diabetic mice. Proc Natl Acad Sci U S A 97: 8015–8020. 10859350 10.1073/pnas.120055097 PMC16662

[45] Vlassara H, Striker LJ, Teichberg S, Fuh H, Li YM, et al (1994) Advanced glycation end products induce glomerular sclerosis and albuminuria in normal rats. Proc Natl Acad Sci U S A 91: 11704–11708. 7972128 10.1073/pnas.91.24.11704 PMC45300

[46] Norata GD, Callegari E, Marchesi M, Chiesa G, Eriksson P, et al (2005) High-density lipoproteins induce transforming growth factor-beta2 expression in endothelial cells. Circulation 111: 2805–2811. 15911702 10.1161/CIRCULATIONAHA.104.472886

[47] Yang CW, Vlassara H, Peten EP, He CJ, Striker GE, et al (1994) Advanced glycation end products up-regulate gene expression found in diabetic glomerular disease. Proc Natl Acad Sci U S A 91: 9436–9440. 7937785 10.1073/pnas.91.20.9436 PMC44827

[48] Bianchi S, Bigazzi R, Campese VM (2010) Intensive versus conventional therapy to slow the progression of idiopathic glomerular diseases. Am J Kidney Dis 55: 671–681. 20097461 10.1053/j.ajkd.2009.11.006

[49] Baigent C, Landray MJ, Reith C, Emberson J, Wheeler DC, et al (2011) The effects of lowering LDL cholesterol with simvastatin plus ezetimibe in patients with chronic kidney disease (Study of Heart and Renal Protection): a randomised placebo-controlled trial. Lancet 377: 2181–2192. 21663949 10.1016/S0140-6736(11)60739-3 PMC3145073

[50] Burton JO, Gray LJ, Webb DR, Davies MJ, Khunti K, et al (2012) Association of anthropometric obesity measures with chronic kidney disease risk in a non-diabetic patient population. Nephrol Dial Transplant 27: 1860–1866. 21965589 10.1093/ndt/gfr574

[51] Reaich D, Price SR, England BK, Mitch WE (1995) Mechanisms causing muscle loss in chronic renal failure. Am J Kidney Dis 26: 242–247. 7611258 10.1016/0272-6386(95)90179-5

